# Quantum Prism—Nano Source of Light with Dispersive Spectrum and Optical Upconversion

**DOI:** 10.3390/nano14151277

**Published:** 2024-07-30

**Authors:** Arturs Medvids, Patrik Ščajev, Saulius Miasojedovas, Kazuhiko Hara

**Affiliations:** 1 Insitute of Technical Physics, Riga Technical University, Paula Valdena 3/7, 1048 Riga, Latvia; 2 Institute of Photonics and Nanotechnology, Faculty of Physics, Vilnius University, Saulėtekio Ave. 3, 10257 Vilnius, Lithuania; saulius.miasojedovas@ff.vu.lt; 3 Research Institute of Electronics, Shizuoka University, 3-5-1 Johoku, Naka-ku, Hamamatsu 432-8561, Japan; hara.kazuhiko@shizuoka.ac.jp

**Keywords:** nanoprism, photoluminescence, light source

## Abstract

A quantum prism, a new structure, consisting of many quantum wires with a diameter that gradually decreases from the base to the top, is the focus of our research. This distribution of quantum wires leads to a dispersive emitted spectrum. The red edge of the spectrum is determined by the band gap width of the bulk semiconductor, and the blue edge is determined by the quantum size of the excitons at the top of the prism. The PL spectrum of the silicon prismatic sample was excited by weak and strong light absorption. At weak absorption (hν_ex_ = 1.2 eV), the PL spectrum is located in the visible part of the spectrum, from 1.4 eV to 1.9 eV, with an energy higher than the band gap of the Si crystal. Such a “blue shift” of PL spectra by 0.7 eV is characteristic of the quantum confinement effect. It is a rainbow spectrum with an optical upconversion. The quantum prism is a new type of nano light source, as it replaces two elements in a conventional spectrometer: a light source and a dispersive element. These features enable to create a nano-spectrometer for measuring the absorption spectrum of individual molecules or viruses.

## 1. Introduction

Nanostructures (NSs) are the most studied objects in solid-state physics, especially the quantum size effect in systems of quantum dots—0D, quantum wires—1D, and quantum wells—2D. Thanks to these conditions, it is possible to create new electronic and optical devices. On the other hand, the tendency to reduce the size of artificial light sources has made it possible to use such sources in microelectronics, medicine, and biology to study micro-objects. At the same time, the requirements for improving the quality of such sources, increasing the intensity, expanding the radiation spectrum, and increasing the uniformity and controllability of its parameters have increased. The importance of this direction is confirmed by the appearance of international conferences and monographs in this direction. Spectroscopic studies of microscopic objects, such as microbes, molecules, and blood cells, require nontraditional research methods, such as an optical probe [1], since the size of such objects is smaller than the wavelength of visible light; therefore, the micro-object does not absorb and scatter it effectively. To study a micro-object, a light source much smaller than the object itself is required, such as quantum dots—0D. Hence, the awarding of the 2023 Nobel Prize in Chemistry to Mungo J. Bawenda, Louis E. Bruce, and Oleksiy I. Yekimov “for the discovery and synthesis of quantum dots” confirms that the quantum confinement effect is a significant focus in solid-state physics today [2]. Therefore, one of the main tasks of scientists is the development of new optical and electronic devices and technologies based on the effect of quantum confinement: quantum dots [3,4] or quantum wires.

We have shown the possibility of the formation of quantum cones [5,6,7]. The quantum cone consists of many points, and the diameter gradually increases from the top to the base of the cone. This distribution of quantum dots leads to a dispersion spectrum. In addition, a change in the diameter of the dots leads to a change in the diameter and energy of excitons [8] and, therefore, their lifetime. This light source is characterized by radiation at a solid angle of 380° and low intensity due to the whole solid angle and the small size of the quantum dot.

The aim of this study is to elaborate a quantum prism as a nano source of light with a dispersive spectrum and optical upconversion. We propose a new light source, a quantum nanoprism, and investigate its photoluminescence properties for applications in nano-spectrometers.

## 2. Theory and Materials

**Figure 1:**
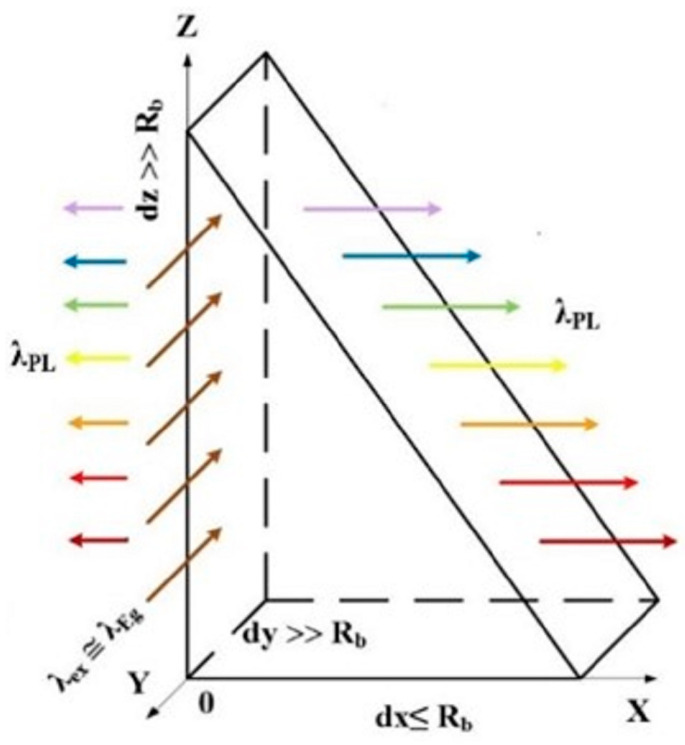
Geometric scheme of a quantum prism with quantum confinement in the OX direction and without it in the OY and OZ directions. Irradiation of the prism is in OY and photoluminescence in the OX directions, respectively.

The quantum nanoprism is schematically shown in Figure 1. The radiation intensity of the quantum prism is higher than that of the quantum cone [5,6,7] due to the limit dy ≤ R_B_ (R_B_ is the Bohr’s exciton radius) and without limitations in the OY direction. This is the proportional relationship between the length of the quantum prism in the OY direction and the diameter of the quantum dot at the same point. Also, the radiation of a quantum prism occurs at a full angle of 180°. In addition, irradiation of the prism in the OY direction by light with low absorption λ_ex_ ≤ λ_Eg_ leads to the formation of excitons with gradually decreasing energy from the top of the prism to its base. Since the bandgap E_g_ in the OZ direction gradually decreases from the top of the prism to its base, according to the modified formula for Si, the ∆E_g_ function on the diameter of the nanowire is calculated [9].

∆E_g_ = (2Ћ^2^ζ^2^)/(m*d_(OZ)_^2^) (1)

where 1/(m*) = 1/(m_e_*) + 1/(m_h_*), m_e_* and m_h_* are electron and hole effective masses, respectively, and *d* is the nanowire diameter. For QWs, *ζ* = 2.4048. In our case, the diameter of the nanowires is a function of the height d(OZ). Thus, it is a semiconductor structure with a graded band gap. First of all, we have a light source with a dispersion spectrum in the OX direction from hν_max_ = E_g max_ to hν_min_ = E_go_, where E_go_ is the band gap of the bulk semiconductor. Secondly, since the energy of the exciting light quantum is less than E_go_, anti-Stokes photoluminescence (ASPL), the so-called optical upconversion should be observed [10,11]. The upconversion phenomenon benefits many applications, such as solar cells, to improve efficiency [12], condensed-phase optical cooling [13], etc.

**Figure 2:**
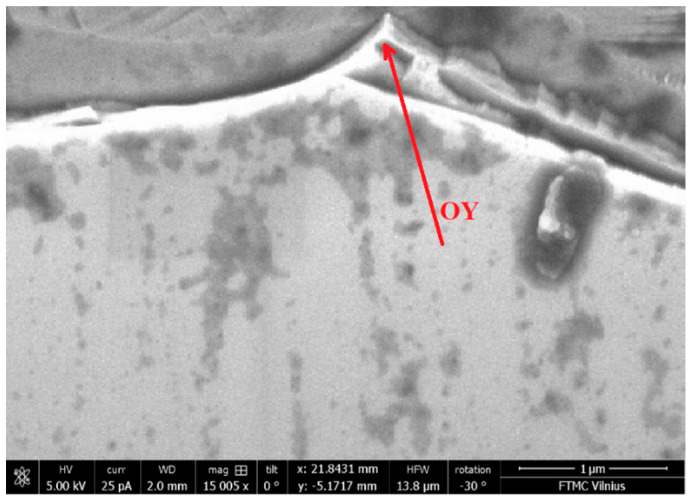
SEM microscope image of SOI sample with nanoprism. The arrow shows the excitation spot.

The experiments were carried out on micro-cutting of an SOI (silicon on insulator) (110) structure with the following parameters: top Si thickness 200 nm, box 400 nm, resistivity 20–30 Ω·cm, doped by boron. After cutting the structure, we chose a prism with an angle at the top of the prism less than 60°. An SEM microscope image of the SOI sample with nanoprism is shown in Figure 2. All experiments were carried out at room temperature.

The photoluminescence (PL) spectra were measured using a Pharos Light Conversion laser coupled with an optical parametric amplifier via an Orpheus module. The width of the laser pulse was approximately 160 fs, and the intensity was up to 1 GW/cm^2^. Light from the sample was collected via an Acton monochromator and directed to the Hamamatsu streak camera. Excitation of PL in the SOI sample with strong hν_ex2_ = 2.1eV and weak hν_ex1_ = 1.2 eV light absorptions for silicon was carried out in the OY direction, and the PL spectra were measured in the OX direction, as shown in Figure 1.

## 3. Results and Discussion

**Figure 3:**
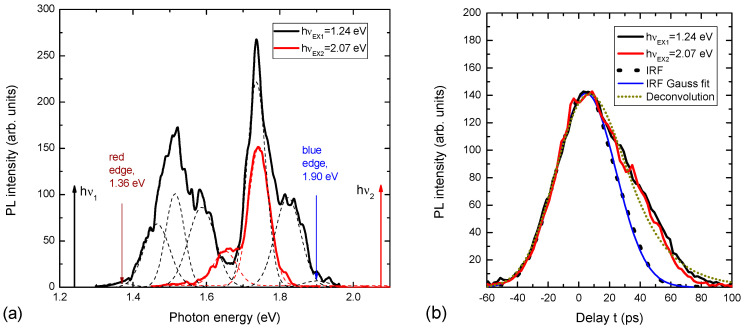
(**a**) PL spectra of quantum prism formed on SOI and excited with strong silicon absorption at hν_ex2_ = 2.1 eV (red curve), and weak silicon absorption hν_ex1_ = 1.2 eV (black curve). (**b**) The corresponding PL decays and system instrumental response function (dashed line with Gauss fit of 40 ps FWHM).

The measured PL spectra of the nanoprism are shown in Figure 3. The PL spectrum for strong absorption by hν_ex2_ = 2.1 eV is atypical for a Si crystal because it is situated at an energy higher than the band gap of Si; a “blue shift” to 1.5 eV takes place. It is an optical downconversion.

The PL spectrum with weak absorption light at hν_ex1_ = 1.2 eV is broader than strong absorption with four maxima and shoulders. Higher PL intensity and broad spectrum, compared to strong absorption, are due to the volume absorption of light by the prism [14]. More maxima and shoulders in the PL spectra are explained by the inhomogeneous prism or the presence of the addition of tiny prisms, as can be seen in Figure 2. Moreover, the PL spectrum is continuous from red to green color. It is an optical upconversion with a rainbow-like spectrum. This means that there is the possibility of upconverting excitation light with controllable energy of light quanta. It depends on the excitation place of the prism; for example, if the excitation takes place at the top of the prism, the energy light quanta will be maximal, and when it goes down to the base of the prism (in the OY direction), the energy of light quanta will decrease. This infrared absorption is important, as prism excitation could be achieved inside organism tissues due to their transparency to infrared.

The study of PL kinetics (PL(t)) showed that the decay time is ~20 ps. The lifetime values were determined by deconvolution of the decays using the system response function IRF = const × exp(−t^2^/2σ^2^), σ = (20 ± 2) ps (Figure 3b) and fitting the function PL(t)~exp[(σ^2^ − 2tτ)/(2τ^2^)] × [1 + Erf{(tτ − σ^2^)/(2^1/2^στ)}] [15], where τ is the decay lifetime. The determined τ value of (21 ± 2) ps was fitted for both high and low excitation quanta. The decay time did not depend on the emission wavelength. In the experiment, the SiO_2_ layer is also irradiated. Therefore, it is necessary to consider this possibility as well. The PL spectrum of the SiO_2_ layer is located in the visible part of the spectra at hν = 1.9 eV [16,17], but it is having usually one band, and the PL decay time is in the range of several milliseconds. The supposed mechanism of upconversion is in two steps: the first creates a cold population of free electrons and holes using a light tuned to the Si bandgap, and the second subsequent phonon coupling results in the carrier with higher energy than the incident light, a so-called Pringsheim mechanism [18]. Evidence of this idea is investigated, for example, by upconversion in shell–core quantum structures [19].

**Figure 4:**
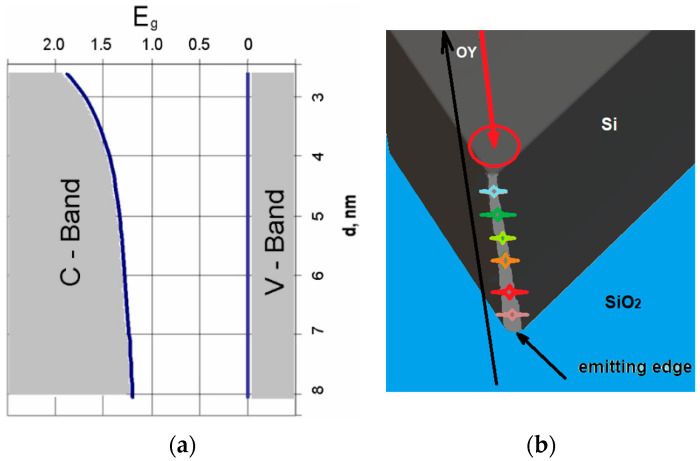
(**a**) Calculation of E_g_ along the height of the prism in the direction of OZ, using PL spectrum red and blue edge peak energies (for black curve in Figure 3) and Formula (1). (**b**) Schematic prism top roundness dependence on its thickness and its correlation with nanowire shortest emission wavelength, shown by different color. The red circle shows the excitation spot. The laser beam excites the prism in the OY direction.

The calculation of the change in E_g_ as a function of height in the OZ direction using the spectrum in Figure 3 (“blue edge” position) and Formula 1 and silicon parameters (the range for d variation was determined from the red and blue edges in Figure 3a) is shown in Figure 4a. It can be seen that this prism is a graded bandgap with a gradual decrease in E_g_ from the top of the prism to its base only due to the quantum size effect in quantum wires. The most-emitting places of the prism are localized on the sharp prism top. The prism top dimensions according the curve in Figure 4a and shortest emitted quanta in Figure 3 are in the range of 2.5–4 nm, and are determined by micromachining precision affecting the prism sharpness. If we take a section of a nanoprism with 60° triangles with a base of 8 nm, this is the diameter of the lowest nanowire in the nanoprism in which there is the weakest QCE, as taken from Figure 4a. The indirect bandgap of the Si crystal is E_go_ = 1.1 eV, and the height of the active part of the nanoprism will be only 7 nm. Eight Gaussian peaks were fitted with different peak emission wavelengths to describe the PL spectra in Figure 3a. They correspond to approximately six dominant nanowires located on the prim top along its edge, and their shortest emission wavelength is shown in Figure 4b. The nanowires of shorter emission wavelength are located closer to the sample surface and are the shortest ones as the prism top radius increases towards its interface with SiO_2_. This means that the quantum prism is a nano source of light with a dispersion spectrum [6].

Such a nano source of light is usable for the study of small medical and biological objects, such as organelles, microbes, and viruses.

## 4. Conclusions

This study shows the possibility of constructing a nano source of light, a quantum prism with a dispersive spectrum distributed along the prism height. The range of the dispersive spectrum depends on the material of the quantum prism and its parameters. The red edge of the spectrum is determined by the band gap of the bulk semiconductor, and the blue edge by the quantum confinement of electron–hole pairs/excitons on top of the quantum prism.

The possibility of upconverting light with a controllable energy of light quanta is shown. This quantum prism is a new type of nano source of light because it substitutes for two elements in a conventional spectrometer: a source of light and a dispersive element.

## Data Availability

The data that support the findings of this study are available upon reasonable request from the authors.
